# Familial lupus associated with a *P2RY8* variant: Navigating the boundary between monogenic disease and genetic susceptibility to lupus

**DOI:** 10.70962/jhi.20260007

**Published:** 2026-03-27

**Authors:** Clémence David, Anne Welfringer-Morin, Luis Seabra, Yanick J. Crow, Marie Jachiet, Marie-Louise Frémond

**Affiliations:** 1 Université Paris Cité, Imagine Institute, Laboratory of Neurogenetics and Neuroinflammation, INSERM UMR 1163, Paris, France; 2 Department of Internal Medicine, Hôpital Bichat-Claude Bernard, Assistance Publique Hôpitaux de Paris, Université Paris Cité, Paris, France; 3 Department of Paediatric Dermatology, Assistance Publique Hôpitaux de Paris, Necker University Hospital, Paris, France; 4 MRC Human Genetics Unit, Institute of Genetics and Cancer, University of Edinburgh, Edinburgh, UK; 5 Dermatology Department, Assistance Publique Hôpitaux de Paris, Saint Louis Hospital, Université Paris Cité, Paris, France; 6 Paediatric Haematology-Immunology and Rheumatology Unit, Necker Hospital, Assistance Publique Hôpitaux de Paris, Université Paris Cité and Reference Centre for Inflammatory Rheumatism, Autoimmune Diseases and Systemic Interferonopathies in Children, Paris, France

## Abstract

Identification of a heterozygous P2RY8 E323G substitution in a father and son with cutaneous lupus and enhanced type I interferon signaling supports a role for P2RY8 in lupus pathogenesis and highlights the overlap between Mendelian disease and complex susceptibility.

## Teaser

Identification of a heterozygous P2RY8 E323G substitution in a father and son with cutaneous lupus and enhanced type I interferon signaling supports a role for P2RY8 in lupus causation and highlights the overlap between Mendelian disease and complex genetic susceptibility.

Systemic lupus erythematosus (SLE) encompasses a spectrum of autoimmune phenotypes, ranging from isolated cutaneous involvement to multi-organ systemic disease (1). Lupus pathogenesis is complex and multifactorial. However, two key processes involved are the activation of the type I interferon (IFN) signaling pathway, and the production of autoantibodies targeting nucleic acids and nucleic acid–binding proteins (1). The discovery of monogenic causes of lupus has provided new insights enabling a better understanding of the molecular mechanisms driving disease, with mutations in proteins involved in nucleic acid metabolism (2), endosomal Toll-like receptor activation, complement pathway signaling, and B and T cell regulation all implicated in lupus pathogenesis.

In 2022, He et al. reported three germline pathogenic variants in the purinergic receptor gene *P2RY8*, encoding the G protein–coupled receptor P2RY8, in patients presenting with SLE or antiphospholipid syndrome, highlighting the role of P2RY8 in B cell homeostasis within germinal centers (3). Here, we describe a familial example of lupus affecting a father and his son, both carrying the previously published heterozygous E323G *P2RY8* variant. We propose that this variant contributed to disease development in this family, and discuss how this finding illustrates the overlap between highly penetrant monogenic SLE, and a more generalized genetic predisposition to the disease.

**Figure 1.**
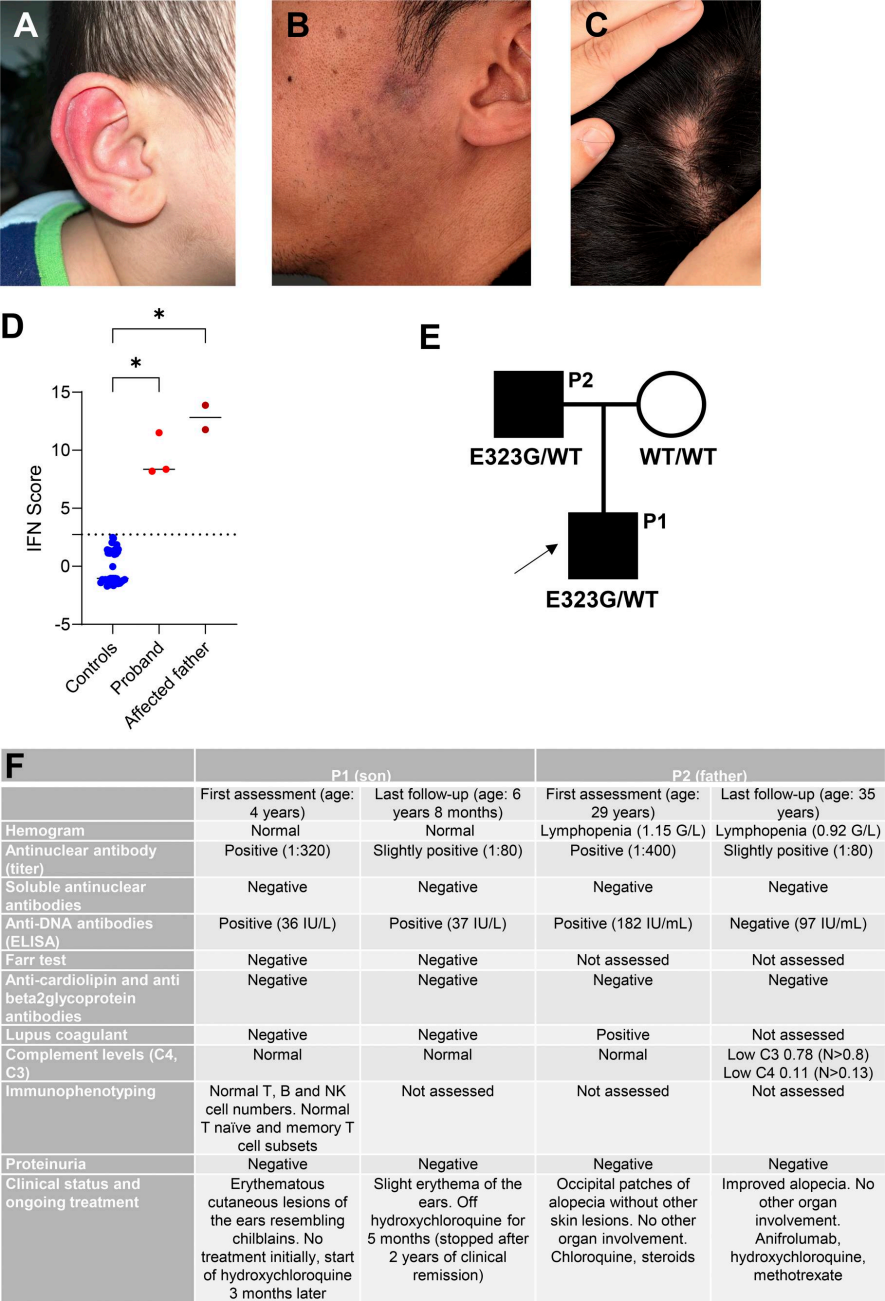
**Cutaneous involvement and type I** **IFN** **signaling status in the two affected patients. (A)** Erythematous cutaneous lesions of the ears resembling chilblains in the son at age 5 years. **(B)** Infiltrated violaceous plaques in the father at age 35 years. **(C)** Alopecic patches in the father at age 35 years. **(D)** Type I IFN pathway activation in the proband and his father harboring the *P2RY8* variant assessed through the analysis of the expression of 24 IFN-stimulated genes and 3 housekeeping genes using the NanoString technology. The horizontal bar indicates the median. Kruskal–Wallis test, *P < 0.05. Dotted lines indicate the upper control values of 2.724. **(E)** Family pedigrees where an affected individual harbors the heterozygous E323G missense substitution in P2RY8. Circles and squares indicate female and male family members, respectively. Filled shapes indicate affected status. The arrow indicates the proband. WT: wild type. **(F)** Table recapitulating the clinical phenotype of the two affected patients.

The proband is a previously healthy young boy of Vietnamese origin, born at term (38 wk gestation, birth weight 3.45 kg) after an uneventful pregnancy, who presented at age 4 years with chronic, recurrent erythematous cutaneous lesions of the ears resembling chilblains without seasonal association (Fig. 1 A). Over time, the child developed additional edematous and erythematous skin lesions on the knees and elbows evocative of lupus tumidus. The clinical examination was otherwise normal, as was testing of urine and full blood cell count. Autoantibody assessment identified positive antinuclear antibodies (ANA) at a titer of 1:320, and positive anti-double-stranded DNA (anti-dsDNA) antibody titers. A 24-gene IFN signature, assessed using the NanoString technology, was markedly elevated on three separate assessments over a period of 18 months (Fig. 1 D). The patient was diagnosed with chilblain lupus and started on hydroxychloroquine at the age of 5 years, with subsequent remission of skin features. At last follow-up, 5 months after discontinuation of hydroxychloroquine, he had not developed any new cutaneous lesions (Fig. 1 F).

His father presented at age 25 years with SLE-associated cutaneous involvement (infiltrated violaceous plaques on the nose, ear concha, and preauricular area [Fig. 1, B and F], with occipital patches of alopecia [Fig. 1 C]), lymphopenia (0.97 gram [G]/L), and thrombocytopenia (95 G/L). Titers of ANA and anti-dsDNA antibodies were positive, and he was hypocomplementemic. A 24-gene IFN signature was markedly elevated on two separate occasions taken 3 months apart (Fig. 1 D). Initial treatment included hydroxychloroquine and oral corticosteroids, with subsequent resolution of systemic features. However, he experienced multiple relapses of his cutaneous lupus, leading to the addition of methotrexate. Despite treatment, he had ongoing disease activity with cicatricial alopecia, resulting in the introduction of the anti-IFNAR monoclonal antibody anifrolumab with subsequent improvement of his alopecia (Fig. 1 F).

Given the familial occurrence of cutaneous lupus and the persistent upregulation of IFN signaling in both affected individuals, whole-exome sequencing was undertaken under the hypothesis of a monogenic type I interferonopathy. This analysis revealed a heterozygous variant in the *P2RY8* gene p.Glu323Gly (c.968A>G, E323G) shared between the father and son (Fig. 1 E). This variant is reported at low frequency in gnomAD v4 (51/1,613,010 alleles), mostly in individuals of Asian ancestry, and is predicted as mildly pathogenic according to different *in silico* scores (Combined Annotation Dependent Depletion (CADD) 19, Sorting Intolerant From Tolerant (SIFT) deleterious, PolyPhen-2 possibly damaging). Importantly, this variant has been previously reported as conferring a loss of function *in vitro* in association with SLE (3). No other heterozygous relevant variants were found to be shared by both father and son in an in-house list of ∼500 IFN-related genes (except for heterozygous variants in *ERBIN*, *IFIT5*, *PCDH12*, *PSMG4*, and *SLC37A4*, which either have been associated with human disease only in the context of autosomal recessive inheritance, or have never been linked to any phenotype).

Determining whether the E323G variant is causative as a highly penetrant allele, is coincidental, or represents a risk allele is challenging. Unlike two other P2RY8 substitutions (N97K and L257F) described by He et al., both being ultra-rare and associated with loss of function, the E323G variant was shown to confer a milder *in vitro* impact. In addition, the relatively high frequency among individuals of Asian descent, notable given the Vietnamese ethnicity of the family presented here, raises the possibility that E323G contributes to an increased genetic susceptibility to lupus in this population. The milder *in vitro *effect of the E323G variant, the skin-limited phenotype in the child, the adult onset in the father, and the occurrence of the variant in asymptomatic individuals in the general population highlight the likelihood that additional genetic or environmental factors are required for the development of the lupus phenotype. Still, the cosegregation of the variant with lupus in two generations, both showing elevated IFN signatures and autoantibody positivity, in the absence of another molecular diagnosis, supports a role in causation.

Mechanistically, P2RY8 encodes a G protein–coupled receptor predominantly expressed in germinal center B cells, where it limits B cell migration and proliferation. Loss-of-function variants lead to unrestrained B cell activation, impaired central tolerance, and increased autoreactive B cells (3). These findings were recapitulated in a lupus-prone mouse model expressing P2RY8, in which the authors showed that P2RY8 promotes B cell–negative selection that was abrogated with the expression of the L257F variant (3). As B cells are key producers of nucleic acid–containing immune complexes, their unrestrained activation creates a pro-inflammatory environment leading to endosomal TLR7 and TLR9 activation, which in turn drives type I IFN production, a hallmark of lupus pathogenesis. Of note, P2RY8 is expressed in T cells and myeloid cells, which may also play a pathogenic role in disease causation. In our patients, chronically elevated IFN scores suggest that P2RY8 dysfunction contributes to an IFN-mediated inflammatory disease, consistent with certain monogenic type I interferonopathies. Notably, this is the first report documenting type I IFN activation in lupus in patients harboring *P2RY8* variants. This finding suggests that the use of therapies specifically targeting type I IFN might be useful in this context, with anifrolumab having been recently initiated in the father reported here.

Distinguishing between monogenic lupus and a genetic predisposition remains a major challenge in clinical practice and research. Monogenic lupus is typically defined by high-penetrance variants in single genes that are sufficient to cause disease. Currently around 7% of cases of pediatric-onset lupus are thought to have a monogenic basis (4), while the majority of cases of SLE, even those with very early onset, are considered to arise due to a combination of common genetic variants of small effect and environmental factors. However, the dichotomy between Mendelian diseases and genetic predisposition is becoming increasingly blurred. This situation is exemplified by other lupus-associated variants such as the V117L variant in UNC93B1, which, although considered pathogenic, is present at low frequency in the general population (17/1,612,954 in gnomAD V4), particularly in Asian cohorts (9/16,924 in a local database) (5). These findings challenge the strict Mendelian vs. polygenic distinction in lupus, highlighting the need to consider “monogenic risk alleles” in the genetic analysis of patients that may require additional genetic or environmental hits to manifest clinically.

This case also highlights the importance of genetic screening in early-onset lupus. Pediatric lupus, particularly when it presents in the context of a family history and/or with markedly enhanced type I IFN signaling, is more likely to have a monogenic or oligogenic basis. In clinical practice, genetic findings can clarify the diagnosis for a patient and inform relevant therapeutic approaches. As the cost falls, and accessibility to sequencing technologies increases, incorporating genetic testing into the diagnostic workup of early-onset and familial lupus cases should be considered.

In summary, our study strengthens the association between P2RY8 dysregulation and lupus pathogenesis, notably in dominantly inherited cases of cutaneous lupus with sustained type I IFN activation. The familial cosegregation of the E323G variant with clinical and immunological features of lupus supports its role as a monogenic risk factor. This case highlights the value of integrating clinical phenotyping, IFN pathway assessment, and genetic analysis to better understand the full spectrum of early-onset and familial lupus, and to improve personalized precision care in affected patients.

## Data Availability

The data underlying Fig. 1 are available in the published article and from the corresponding author upon request.
